# Genomic selection of purebreds for crossbred performance

**DOI:** 10.1186/1297-9686-41-12

**Published:** 2009-01-15

**Authors:** Noelia Ibánẽz-Escriche, Rohan L Fernando, Ali Toosi, Jack CM Dekkers

**Affiliations:** 1 Genètica i Millora Animal- Centre IRTA Lleida, 25198 Lleida, Spain; 2 Department of Animal Science, Iowa State University, Ames 50011-3150, USA

## Abstract

**Background:**

One of the main limitations of many livestock breeding programs is that selection is in pure breeds housed in high-health environments but the aim is to improve crossbred performance under field conditions. Genomic selection (GS) using high-density genotyping could be used to address this. However in crossbred populations, 1) effects of SNPs may be breed specific, and 2) linkage disequilibrium may not be restricted to markers that are tightly linked to the QTL. In this study we apply GS to select for commercial crossbred performance and compare a model with breed-specific effects of SNP alleles (BSAM) to a model where SNP effects are assumed the same across breeds (ASGM). The impact of breed relatedness (generations since separation), size of the population used for training, and marker density were evaluated. Trait phenotype was controlled by 30 QTL and had a heritability of 0.30 for crossbred individuals. A Bayesian method (Bayes-B) was used to estimate the SNP effects in the crossbred training population and the accuracy of resulting GS breeding values for commercial crossbred performance was validated in the purebred population.

**Results:**

Results demonstrate that crossbred data can be used to evaluate purebreds for commercial crossbred performance. Accuracies based on crossbred data were generally not much lower than accuracies based on pure breed data and almost identical when the breeds crossed were closely related breeds. The accuracy of both models (ASGM and BSAM) increased with marker density and size of the training data. Accuracies of both models also tended to decrease with increasing distance between breeds. However the effect of marker density, training data size and distance between breeds differed between the two models. BSAM only performed better than AGSM when the number of markers was small (500), the number of records used for training was large (4000), and when breeds were distantly related or unrelated.

**Conclusion:**

In conclusion, GS can be conducted in crossbred population and models that fit breed-specific effects of SNP alleles may not be necessary, especially with high marker density. This opens great opportunities for genetic improvement of purebreds for performance of their crossbred descendents in the field, without the need to track pedigrees through the system.

## Introduction

One of the main limitations of many livestock breeding programs is that selection is in purebred nucleus lines or breeds that are housed in high-health environments but the goal of selection is to improve crossbred performance under field conditions. Due to genetic differences between purebreds and crossbreds and environmental differences between nucleus and field conditions, performance of purebred parents can be a poor predictor of performance of their crossbred descendants [1]. Furthermore, some important traits such as disease resistance cannot be measured in nucleus lines. In order to avoid these problems, it has been proposed to select purebred relatives based on crossbred performance using combined crossbred and purebred selection or CCPS [2-6]. This approach can increase response to selection for crossbred performance relative to the classical method of selection on purebred performance [7]. It has, however, not been extensively implemented in livestock due mainly to the difficulty and cost of routine collection of phenotypic and pedigree data from crossbreds in the field [1]. In addition, using CCPS increases the rate of inbreeding [8] and makes it difficult to accommodate non-additive gene action [6]. As an alternative to CCPS, Dekkers [1] proposed to select purebreds for commercial crossbred performance using genomic selection.

In livestock, genomic selection is becoming increasingly feasible because of the availability of massive numbers of single nucleotide polymorphism (SNP) markers. This approach consists of predicting breeding values on the basis of a larger number of SNPs [9-11], utilizing linkage disequilibrium (LD) between SNPs and the QTL. Genomic selection of purebreds for crossbred performance involves estimating effects of SNPs on crossbred performance, using phenotypes and SNP genotypes evaluated on crossbreds, and applying the resulting estimates to SNP genotypes obtained on purebreds (Dekkers 2007). Genomic selection for crossbred performance has three main advantages over CCPS: 1) it does not require pedigree information on crossbreds, 2) after estimates of SNP effects are obtained using genotype and phenotype data, prediction can continue for several generations without additional phenotypes [9], 3) it reduces the rate of inbreeding [12], and 4) it makes accommodating non-additive gene action easier [1]. The success of genomic selection depends mainly on the prediction accuracy of the estimated breeding values (GEBVs). Several authors have studied the accuracy of these predictions by computer simulation [9,13,14]. However, these studies have focused on pure breeds. In crossbred populations, effects of SNPs may be breed specific because the extent of LD between SNPs and QTL can differ between breeds. Moreover, the LD may not be restricted to markers that are tightly linked to the QTL. Both these problems could be addressed by using a model with breed-specific effects of SNP alleles. Toosi et al. [15] evaluated simulated training populations consisting of crosses or mixtures of breeds and found the accuracy of genomic selection to be lower compared to using purebred data for training, but not by a large degree. They, however, used a genomic selection model in which SNP allele effects were assumed the same in all breeds. Thus, the objective of this study was to compare by computer simulation the accuracy of genomic selection of purebreds for commercial crossbred performance, using either the classical genomic selection model with across-breed effects of SNP genotypes (ASGM) or a model with breed-specific effects of SNP alleles (BSAM).

## Methods

### Simulation

In all simulations, the genome consisted of one chromosome of 1 Morgan with 6000 SNPs and 30 biallelic QTL. A gamma distribution with shape and scale parameters equal to 0.4 and 1/1.66 was used to sample the absolute value of effects of the QTL. The sign of the QTL effect was sampled to be positive or negative with probability 0.5. Effects were rescaled to result in a genetic variance equal to 1.0. The phenotypic trait was simulated under additive gene action. Dominance and epistatic effects were not simulated but would be captured to the extent that they are incorporated in allele substitution effects (see discussion).

In the base population, SNP and QTL alleles were sampled from a Bernoulli distribution with frequency 0.5. A mutation rate of 2.5 × 10^-5 ^per generation was applied in the following generations for all loci, where mutations switched the allele state from 1 to 2 or from 2 to 1. Recombinations on a chromosome were modeled according to a binomial map function [16].

Three scenarios for breed history were considered in this study. In the first two scenarios, the breeds were assumed to have a common origin either 50 or 550 generations ago. In the third scenario, the breeds did not have a common origin. These scenarios will be referred to as having closely related breeds, distantly related breeds, and unrelated breeds, respectively. In all cases LD was simulated by drift and mutation in two periods. In the first period of 1000 generations, random mating was simulated in an effective population of size 500. In the second period of 50 generations, random mating continued after reducing the effective population size to 100. In generation 1051 the population size was expanded to 1000 or 4000 individuals simulating more matings and seven more generations of random mating with the expanded population size were produced. Also, in generation 1051 three different commercial crossbred lines were generated with 1000 or 4000 individuals. These crossbred lines were an AxB two-breed cross, an ABxC three-breed cross, and an ABxCD four-breed cross. The crossbred lines in generation 1051 were used for "training" with phenotype and genotype data, and the purebred lines in generation 1058 for validation with only genotype data. Either 500 or 2000 segregating SNPs (minor allele frequency > 0.05) from the crossbred population were chosen for analysis. Some of these segregating SNPs in the crossbred populations were fixed in the purebred populations. Heritability of the quantitative trait was set to 0.3 by rescaling QTL effects in the training population. The method to estimate SNP effects was Bayes-B [9], which is described further in the following. The criterion to compare models was the accuracy of estimated breeding values for the purebred validation population, calculated as the correlation between true and estimated breeding values. Each simulated data set and analysis was replicated 40 times.

### Statistical Models

The statistical models used for the analyses are described here. The across-breed SNP genotype model (ASGM) is:

(1) $$y_{i}=\mu +\sum_{j}X_{ij}\beta_{j}\delta_{j}+e_{i},$$

where *y*_*i *_is the phenotype of *i*, *μ *is the overall mean, *X*_*ij *_(0, 1, or 2) is the genotype of *i *at marker locus *j*, *β*_*j *_is the across-breed allele substitution effect of locus *j *in the training population, *δ*_*j *_is a 0/1 indicator variable that specifies if locus *j *is included in the model or not, and *e*_*i *_is the residual of *i*. The breed-specific SNP allele model (BSAM) is:

(2) $$y_{i}=\mu +\sum_{j}(A_{ijk}^{S}\beta_{jk}^{S}\delta_{j}^{S}+A_{ijl}^{D}\beta_{jl}^{D}\delta_{j}^{D})+e_{i},$$

where $A_{ijk}^{S}$ (0,1) is the SNP allele at locus *j*, of breed origin *k *that *i *received from its sire, $\beta_{jk}^{S}$ is the breed-specific substitution effect for allele $A_{ijk}^{S}$. If the sire of *i *is a purebred, *k *takes the same value for all alleles, e.g. *k *= 1 if the sire is purebred A. On the other hand, if the sire is crossbred, AxB for example, *k *can take values 1 or 2, indicating whether the SNP allele received for the sire originated from breed A or B.

The variable, $\delta_{j}^{S}$, is a 0/1 indicator that specifies if the sire allele is included in the model for locus *j*.

Similarly, $A_{ijl}^{D}$, $\beta_{jl}^{D}$, and $\delta_{j}^{D}$ are defined for the SNP allele at locus *j*, of origin *l *that *i *received from its dam. Breed origin of alleles was assumed to be known without error in the analyses.

The Bayes-B method described by Meuwissen et al. [9] was used to estimate the across-breed additive effects in ASGM and the breed-specific additive effects in BSAM. The prior probability for a locus to be included in the model was set to 0.05, i.e., Pr(*δ*_*j *_= 1) = 0.05. A previous study of prior sensitivity was performed to validate that it did not influence in the model results. For loci in the model, the locus effects were assumed to be normal with null mean and locus specific variance $\sigma_{\beta_{j}}^{2}$ in ASGM, and locus and breed-origin specific variance $\sigma_{\beta_{jk}^{S}}^{2}$ and $\sigma_{\beta_{jl}^{D}}^{2}$ for BSAM. Following Meuwissen et al. [9], the prior for these variance components was an inverse chi-square with 4.234 degrees of freedom and scale parameter *S *= 0.0429. The prior for the $\sigma_{e}^{2}$ was an inverse chi-square distribution with four degrees of freedom and scale parameter *S *= 0.4, and a flat prior was used for *μ*. A difference between the Bayes-B implementation of Meuwissen et al. [9] and that used here is that we fitted effects of SNP genotypes and alleles rather than of haplotypes. After some exploratory analyses, a single chain of 100,000 samples was used, with a burn-in period of 1000. Convergence was tested for all dispersion parameters separately using the Raftery and Lewis [17] method and a visual check of the chain plots.

## Results

Accuracy of prediction of breeding values in the purebred lines using ASGM and BSAM are in Tables 1, 2 and 3. Results when the AxB two-breed cross was used as training population are in Table 1. In this table, the accuracy of both models (ASGM and BSAM) increased with marker density and size of the training data. Accuracies of both models also tended to decrease with increasing distance between breeds. The effect of marker density, training data size and distance between breeds, however, differed between the two models, which resulted in the model with the highest accuracy to differ between scenarios. Given the differences in marker-QTL LD, we would have expected the model that fitted breed-specific SNP allele effects (BSAM) to have greater accuracy. However, that was the case only when the number of markers was small (500), the number of records used for training was large (4000), and when breeds were distantly related or unrelated. When the number of markers was increased to 2000, ASGM gave better results when breeds were closely related, and the difference in accuracy was significant in the simulation with 1000 records. For distant or unrelated breeds, BSAM had accuracies that were equal to or better than those with ASGM.

**Table 1:** Accuracy (se) of breeding values in pure breed predicted based on two-breed cross data using ASGM or BSAM for three different scenarios (40 replicates)

		closely related breeds			distantly related breeds			unrelated breeds		
1000 records										

Markers	VP^a^	ASGM	BSAM	Diff^b^	ASGM	BSAM	Diff^b^	ASGM	BSAM	Diff^a^

500	B	0.78	0.79	-0.01	0.72	0.76	-0.04	0.72	0.73	- 0.02
		(0.01)	(0.02)	(0.01)	(0.05)	(0.04)	(0.02)	(0.03)	(0.03)	(0.01)
2000	B	0.87	0.81	0.06	0.81	0.81	0.00	0.80	0.81	-0.01
		(0.01)	(0.01)	(0.01)	(0.01)	(0.01)	(0.01)	(0.01)	(0.01)	(0.01)

4000 records										

Markers	VP^a^	ASGM	BSAM	Diff^b^	ASGM	BSAM	Diff^b^	ASGM	BSAM	Diff ^b^

500	B	0.83	0.85	-0.02	0.78	0.82	-0.04	0.77	0.80	-0.03
		(0.01)	(0.01)	(0.01)	(0.02)	(0.02)	(0.01)	(0.03)	(0.03)	(0.01)
2000	B	0.92	0.91	0.01	0.91	0.91	0.01	0.88	0.91	-0.03
		(0.01)	(0.01)	(0.01)	(0.01)	(0.01)	(0.01)	(0.02)	(0.02)	(0.01)

^a ^Pure breed used as validation population.

^b ^Difference (se) of accuracy between ASGM and BSAM.

**Table 2:** Accuracy (se) of breeding values in pure breed predicted based on three-breed cross data using ASGM or BSAM for three different scenarios (40 replicates)

		closely related breeds			distantly related breeds			unrelated breeds		
1000 records										

Markers	VP^a^	ASGM	BSAM	Diff^b^	ASGM	BSAM	Diff ^b^	ASGM	BSAM	Diff^b^

500	B	0.68	0.63	0.05	0.57	0.59	-0.02	0.44	0.42	0.02
		(0.02)	(0.03)	(0.02)	(0.03)	(0.04)	(0.02)	(0.03)	(0.04)	(0.03)
	C	0.79	0.74	0.05	0.64	0.63	0.01	0.56	0.57	-0.02
		(0.02)	(0.02)	(0.01)	(0.03)	(0.03)	(0.01)	(0.03)	(0.03)	(0.02)
2000	B	0.82	0.74	0.08	0.66	0.63	0.04	0.63	0.63	0.00
		(0.02)	(0.02)	(0.01)	(0.04)	(0.04)	(0.02)	(0.02)	(0.02)	(0.01)
	C	0.85	0.73	0.11	0.77	0.68	0.09	0.71	0.67	0.04
		(0.03)	(0.02)	(0.02)	(0.03)	(0.02)	(0.02)	(0.02)	(0.02)	(0.01)

4000 records										

Markers	VP^a^	ASGM	BSAM	Diff^b^	ASGM	BSAM	Diff^b^	ASGM	BSAM	Diff^b^

500	B	0.79	0.81	-0.02	0.68	0.75	-0.07	0.63	0.71	-0.08
		(0.02)	(0.02)	(0.01)	(0.02)	(0.03)	(0.01)	(0.03)	(0.06)	(0.03)
	C	0.82	0.79	0.02	0.74	0.74	0.00	0.76	0.77	0.01
c		(0.02)	(0.02)	(0.01)	(0.03)	(0.03)	(0.01)	(0.03)	(0.03)	(0.05)
2000	B	0.87	0.86	0.01	0.85	0.87	-0.02	0.79	0.67	0.11
		(0.04)	(0.01)	(0.01)	(0.02)	(0.02)	(0.01)	(0.05)	(0.04)	(0.02)
	C	0.92	0.86	0.06	0.83	0.80	0.03	0.79	0.72	0.06
		(0.02)	(0.01)	(0.01)	(0.02)	(0.02)	(0.01)	(0.05)	(0.04)	(0.02)

^a ^Pure breed used as validation population.

^b ^Difference (se) of accuracy between ASGM and BSAM.

**Table 3:** Accuracy (se) of breeding values in pure breed predicted based on four-breed cross data using ASGM or BSAM for three different scenarios  (40 replicates)

		closely related breeds			distantly related breeds			unrelated breeds		
1000 records										

Marker	VP^a^	ASGM	BSAM	Diff^b^	ASGM	BSAM	Diff^b^	ASGM	BSAM	Diff^b^

500	B	0.65	0.60	0.05	0.46	0.48	0.02	0.46	0.50	-0.03
		(0.03)	(0.03)	(0.03)	(0.04)	(0.04)	(0.03)	(0.08)	(0.08)	(0.05)
2000	B	0.84	0.75	0.09	0.62	0.58	0.04	0.52	0.54	- 0.02
		(0.02)	(0.02)	(0.02)	(0.04)	(0.04)	(0.02)	(0.03)	(0.03)	(0.01)

4000 records										

Marker	VP^a^	ASGM	BSAM	Diff^b^	ASGM	BSAM	Diff^b^	ASGM	BSAM	Diff ^b^

500	B	0.78	0.80	-0.02	0.62	0.72	-0.11	0.55	0.70	-0.14
		(0.02)	(0.02)	(0.01)	(0.03)	(0.03)	(0.02)	(0.02)	(0.03)	(0.03)
2000	B	0.87	0.85	0.01	0.85	0.86	-0.01	0.72	0.62	0.10
		(0.01)	(0.04)	(0.02)	(0.03)	(0.02)	(0.01)	(0.05)	(0.05)	(0.02)

^a ^Pure breed used as validation population.

^b ^Difference (se) of accuracy between ASGM and BSAM.

As a reference, accuracy of predicting breeding value in a purebred line when training was in the same line is given in Table 4. These results are almost identical to those in Table 1 for closely related breeds.

**Table 4:** Accuracy of breeding values in pure breed predicted based on performance in the same pure breed using ASGM (40 replicates)

		1000 records	4000 records
Marker	% PB^a^	ASGM	ASGM

500	100%	0.79 (0.02)	0.83 (0.03)
2000	100%	0.91 (0.01)	0.94 (0.01)

^a ^Percentage in the training population of the breed evaluated.

Accuracies of the best model in the cross are, however, lower for distant and unrelated breeds. Results when the ABxC three-breed cross was used as the training population are in Table 2. In this scenario, 50% of the alleles in the training population are from breed C but only 25% are from either breed A or B. Thus, accuracies are given in this table for predicting breeding values of B and C purebred animals. In all cases and for both models, accuracies were lower for breed B than for breed C, as expected. Also, general trends in accuracies for a given model with changes in marker density, data size, and breed distance were similar as observed for the two-way cross in Table 1. The relative performance of the two models, however, differed from what was observed for the two-way cross. For the three-way cross (Table 2), with closely related breeds, ASGM gave better results when 1000 records were used, and with the exception of predicting purebred B animals using 500 markers, all these differences were significant. For close breeds, when 4000 records were used for training, ASGM was significantly better only for predicting purebred C animals using 2000 markers. For distant or unrelated breeds, ASGM was significantly better than BSAM for predicting purebred C animals using 2000 markers and 1000 records for training. When the number of records for training was increased to 4000, BSAM was significantly better for predicting purebred B animals using 500 markers in scenario 2, but ASGM was better for predicting purebred B animals using 500 markers in scenario 3 and for predicting purebred C animals using 2000 markers in scenarios 2 and 3.

**Figure 1:**
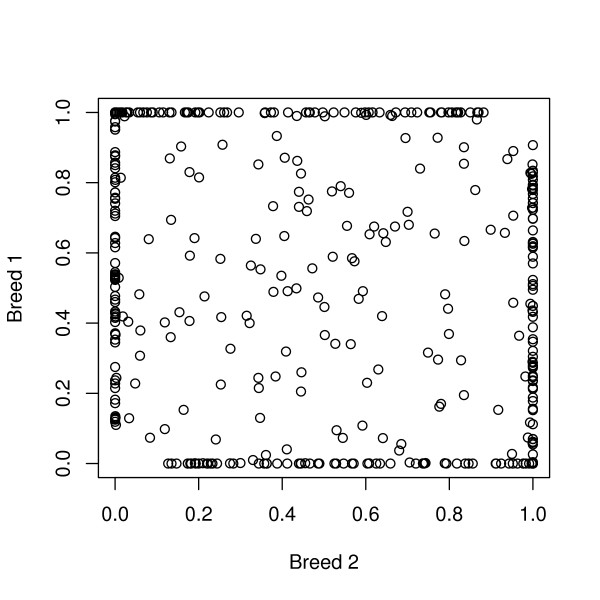
Frequency of SNP alleles for purebreds A and B in generation 1050 for unrelated breeds

Results when the ABxCD four-breed cross was used as the training population are in Table 3. Because the same accuracy is expected for all breeds, since all contribute 25% to the cross, only accuracy for one breed is shown. Here, BSAM was significantly better when 500 markers were used with 4000 records for training for distant or unrelated breeds. However, ASGM was significantly better when 2000 markers were used with 1000 records for training for close breeds and with 4000 records for training for unrelated breeds. Figure 1 shows the frequency of SNP alleles for purebreds A and B in generation 1050 for unrelated breeds. This figure shows that a large number of loci that were segregating in one of the purebred lines were fixed in the other purebred line. For these loci that are fixed in one of the purebred lines, ASGM and BSAM are equivalent. This partially explains why differences between ASGM and BSAM were small for unrelated breeds.

**Figure 2:**
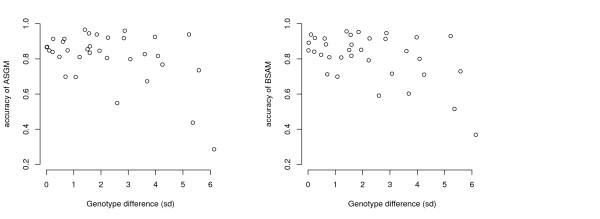
**Difference in average genotypic values of two breeds against the accuracy of breeding values predicted based on their crossbred data. **Each point represents one replicate for the scenario with distantly related breeds, 2000 SNPs, and 1000 records.

To further investigate the impact of the genetic difference between breeds on the accuracy of genomic selection based on crossbred data, Figure 2 plots the difference in average genotypic values of the two breeds against the accuracy of breeding values predicted based on their crossbred data. Each point represents one replicate for the scenario with distantly related breeds, 2000 SNPs, and 1000 records. Although in general high accuracies were obtained for genotype differences smaller than 4 sd, the small number of samples with breed differences greater than 4 sd was not enough to disclose a clear relationship between breed difference and accuracy.

The results presented above were based on a simulated genome consisting of only 1 chromosome of 1 M. To compare these results with a more realistic situation, we simulated the scenario for closely related breeds with a genome of 10 chromosomes with a total genome size of 10 M, 60,000 SNPs and 1,000 QTL. For the statistical analysis we chose 20,000 segregating SNPs from the crossbred population. The analysis of this data showed a 25% drop in of accuracy relative to the results with 1 chromosome. However, the relationship between training in a purebred line or crossbred line did not change (Table 5).

**Table 5:** Accuracy of breeding values in pure breed predicted based on crossbred data when the breeds are closely related for a simulated genome of 10 chromosomes of 1 M each (40 replicates)

1000 records		Training population			
		
		Two-breed cross (AxB)			Purebred B
Marker	VP^a^	ASGM	BSAM	Diff	ASGM

20000	B	0.59	0.54	0.04	0.62
		(0.02)	(0.02)	(0.01)	(0.01)

^a ^Validation population.

## Discussion

The objective of this study was to compare the accuracy of genomic selection of purebreds for commercial crossbred performance using either ASGM or BSAM. Alleles in a crossbred line originate from one of the purebred parental lines. If these purebred lines are not closely related, the effect of SNP alleles will depend on their line of origin. Thus, a model with breed-specific effects of SNP alleles (BSAM) was used to estimate the effects of alleles in purebreds for crossbred performance. These estimated effects and the SNP genotypes of purebred candidates for selection were then used to predict their breeding values for crossbred performance. The accuracy of prediction was quantified by the correlation of the predicted and true breeding values. This accuracy was compared to that obtained using the classical model with across breed effects of SNP genotypes (ASGM).

Due to the genetic differences among the pure lines, BSAM with breed-specific effects of SNP alleles was expected to perform better. Contrary to expectation, however, accuracy of prediction with ASGM often was equal to or higher than with BSAM. In addition to the relationship between the purebred parental lines, there are two other factors that contribute to the difference in accuracy of prediction using ASGM and BSAM in our simulations. Marker density is one of these, and the other is the number of records used in training. Marker density affects the difference between ASGM and BSAM in two ways. The first is that as marker density increases the model will include markers that are closer to the QTL. In a finite population, marker alleles that are closer to the QTL will more accurately reflect the state of the QTL alleles. Thus, as the marker density increases the need for BSAM is reduced. The second is that BSAM has, relative to ASGM, twice as many effects that need to be estimated in a two-breed cross, three times as many in a three-breed cross, and four times as many in a four-breed cross. Thus, due to the greater number of effects that need to be estimated, BSAM is at a disadvantage over ASGM, and this disadvantage increases with marker density. On the other hand, as the number of records used for training increases more information becomes available to estimate the effects of markers and, given sufficient records for training, even small differences in breeds will make BSAM advantageous. So, BSAM will give better results only when breed differences are big enough to compensate for the additional breed-specific effects in the model, given the number of records used for training. Note that in the absence of epistasis, there are no breed differences for effects at the QTL. Thus, as the marker density increases, breed differences of markers effects decreases while the number of extra parameters in BSAM increases.

In Table 1, BSAM had greater accuracy when 500 markers were used, but when the number of markers was increased to 2000, this advantage disappeared except when breeds were unrelated and, thus, breed differences were greatest. The effect of increasing the number of records used for training can be seen from Tables 1, 2, 3, where given the same number of markers, increasing the number of records used tended to favor BSAM. In Table 2, for example, the difference in accuracy between ASGM and BSAM was not significant with 500 markers for distantly related breeds when 1000 records were used for training, but when the number of records for training was increased to 4000, BSAM was significantly more accurate, with the difference in accuracies between ASGM and BSAM changing from 0.02 to -0.11. Our results include several such examples where increasing the number of records favors BSAM (Tables 1, 2, 3), but none that goes in the opposite direction. This demonstrates that BSAM will have an advantage provided sufficient information is available for estimating the additional breed-specific effects.

In livestock, production animals often are either from a three-breed or four-breed cross. When an ABxC three-breed cross was used for training, the accuracy of prediction of purebred C animals was about the same as the accuracy of prediction of purebred B animals with training in an AxB two-breed cross. This is because 50% of the alleles in the ABxC cross are from purebred line C. On the other hand, only 25% of the alleles in ABxC are from purebred line B. Thus, the accuracy of prediction for line B animals was significantly lower. The same was true in a four-breed cross, where only 25% of the alleles in the crossbreds are from any particular parental line. Thus, the accuracy of prediction of purebred B animals with training in an ABxCD cross was similar in accuracy to that for purebred B animals with training in an ABxC cross (Tables 2 and 3). It is interesting that the accuracy of prediction with training in a four-breed cross using 4000 records was about the same as that with training in a purebred line with 1000 records (Tables 3 and 4).

The results in Table 5 show that, given the same number of records used for training, when marker effects from 10 chromosomes were included in the model, the accuracy of prediction dropped. Table 1 showed that when the model included 2000 markers from one chromosome, ASMG was significantly more accurate than BSAM. When the model includes 20,000 markers from 10 chromosomes, the difference in accuracy became smaller but remained significant (Table 5).

Dominance and epistatic effects were not considered in the present study. However, the genomic selection methods for crossbred performance do not require absence of non-additive effects. If non-additive effects are present, the marker effects estimated by the genomic selection methods are allele substitution effects, which incorporate the additive components of dominance and epistatic effects [18]. Thus, by estimating allele substitution effects based on crossbred phenotypes, the effects of purebred alleles will be estimated against the genetic background that they will be expressed in. Thus, genomic selection on SNP effects estimated on crossbred data is equivalent to practicing reciprocal recurrent selection.

The simulation model also assumed absence of genotype by environment interactions. Such interactions could, however, be present when comparing performance in nucleus and field environments and contribute to the low genetic correlations between purebred and crossbred performance that have been estimated in literature. However, similar to non-additive effects, allele substitution effects estimated based on phenotypes collected in the field would allow the effects of purebred alleles to be estimated under the environment in which they will be expressed.

Although genomic selection models accommodate non-additive effects to the extent that they are captured by allele substitution effects, presence of non-additive effects can reduce the accuracy of GEBV compared to those obtained here, and also affect the comparison between the ASGM and BSAM models. The reason is that non-additive effects will increase differences in breed-specific allele substitution effects because breeds are expected to differ in allele frequencies at QTL. Specifically, with dominance, the QTL allele substitution effect for breed A on performance of AxB crossbreds is equal to a+d(1-2pB), where pB is the QTL allele frequency in breed B and a and d are the additive and dominance effects at the QTL [19]. Thus, if breeds that are being crossed have different QTL allele frequencies, they will have different allele substitution effects at the QTL and, therefore also at markers that are in LD with the QTL. Epistatic effects also contribute to allele substitution effects, depending on allele frequencies. Thus, if epistatic effects are present, allele substitution effects will further differ between breeds. These additional differences in breed-specific SNP effects compared to what was simulated here will likely increase the accuracy of the BSAM model that includes breed-specific allele effects compared to the ASGM model. The accuracy of the ASGM model will likely decrease slightly, as the average allele effects across breeds will tend to be reduced when differences in breed-specific allele effects are greater. Further work is needed to investigate these scenarios. Presence of genotype by environment interactions for the nucleus versus field environment are not expected to affect the accuracy of either the ASGM or BSAM model because allele effects are evaluated in the target environment for both models.

In this study, divergence between breeds was created by drift only. In practice, in addition to drift, breeds will have diverged as a result of different selection pressures imposed upon them through either artificial or natural selection. The potential impact of differential artificial selection on the trait being evaluated is indirectly evaluated in Figure 2 by considering breed pairs that have drifted apart to differing degrees for average genotypic values for the trait. As shown in the Figure, this did not have a discernible effect on the accuracy of genomic selection. The same is expected to hold for breeds that have been differentially selected for other characteristics.

Results from this study show the potential for genomic selection of purebreds for commercial crossbred performance. This would enable genetic improvement of purebreds for performance of their crossbred descendents in the field, without the need to track pedigrees through the system. Further, these results indicate that a model with breed-specific effects of alleles may not be necessary, especially when the marker density is high. It is obvious that ASGM would be better when breeds are not very different. However, in some cases ASGM was significantly better even when the breeds did not have any common origin (Table 3). The reason for this can be seen from figure 1. There are three types of loci in this figure: 1) those that are segregating in both lines, 2) those that are segregating only in one line, and those that are fixed in both lines. Loci of the first type would favor BSAM, those of the second type would contribute equally to both models, and those of the third type would not contribute to either. Crosses of highly inbred lines that were separated in the distant past will have only a few loci of the first type and thus, would not favor BSAM over ASGM. So, even in this extreme case, ASGM can do well. Using ASGM has the advantage that it does not require tracing alleles from crossbreds in the field to their purebred ancestors in nucleus lines. In this study, we assumed that alleles could be traced from the crossbreds to the purebred parents without error. Given very high density marker information, it may be possible to trace alleles to ancestors very accurately [20], but some errors may be inevitable. Thus, in practice, ASGM may even perform relatively better than in this study.

## Authors' contributions

NIE participated in the design of the study, carried out the simulation studies, performed the statistical analyses, and drafted the manuscript. AT participated in the design of the study and helped with the simulation studies. RLF and JCMD conceived of the study, oversaw its design and execution, and helped to revise and finalize the manuscript. RLF also assisted with development of the simulation and analysis programs. All authors read and approved the final manuscript.
